# Cerebral Microbleeds in an Acute Ischemic Stroke as a Predictor of Hemorrhagic Transformation

**DOI:** 10.7759/cureus.3308

**Published:** 2018-09-15

**Authors:** Nayab Z Dar, Qurat Ul Ain, Rashed Nazir, Arsalan Ahmad

**Affiliations:** 1 Radiology, Shifa International Hospital, Islamabad, PAK; 2 Medical Officer, Shifa College of Medicine, Shifa International Hospital, Islamabad, PAK; 3 Neurology, Shifa International Hospital, Islamabad, PAK

**Keywords:** hemorrhagic transformation, cerebral microbleeds, ischemic stroke, cerebrovascular disease

## Abstract

Background

Cerebral microbleeds are small, round hypointensities of <10 mm in diameter, evident on T2* gradient-recall echo (GRE) or susceptibility-weighted (SWI) magnetic resonance imaging (MRI) sequences.

Objective

In this study, our objective was to determine the number and location of cerebral microbleeds in ischemic stroke and to identify the predictive role of microbleeds for hemorrhagic transformation.

Materials and methods

This was a retrospective cohort study. Microbleeds were visually rated on SWI scans of patients who presented with an ischemic stroke and had an SWI scan within 24 hours of onset and a computed tomography (CT)/MRI scan during follow up. Microbleeds were graded as Grades I-IV.

Results

Out of 575 stroke patients, 121 did not have an SWI scan and 336 had no follow-up scan. A total of 118 patients were included for a final analysis (75 males, 43 females) out of which 30 had a hemorrhagic transformation. Most microbleeds were in the parietal region (n=46) with 50% transformation (p-value <0.001). The size and grade of microbleeds had a statistical association with hemorrhagic transformation with p-value 0.001 and p-value <0.001, respectively; 33% of patients with Grade 3 microbleeds aging 55-65 years had transformations. Of the patients, 93.3% with Grade 4 microbleeds had a hemorrhagic transformation. 30% of transformations were detected in the first 24 hours while 30% were detected during the first week. Age, gender, comorbidity, and anticoagulant use had no statistical association of conversion of microbleeds into hemorrhagic transformation.

Conclusion

Microbleeds detected on an SWI scan is a relevant and accurate predictor of hemorrhagic transformations in acute ischemic infarcts and should be added to MRI stroke protocols.

## Introduction

The term “cerebral microbleeds,” or “microhemorrhages,” refer to “small, round, or ovoid hypointensities, of <10 mm in diameter, evident on T2* gradient-recall echo (GRE) or susceptibility-weighted imaging (SWI)/magnetic resonance imaging (MRI) sequences.” [1]. Different studies have indicated that susceptibility-weighted MRI sequences are more sensitive in detecting hemorrhages inside acute infarct lesions, as they show more clear images of microbleeds instead of CT and 2D gradient recalled-echo (2D-GRE) T2-weighted imaging [2].

A prospective blinded randomized trial has proved that SWI could detect hemorrhagic transformation even a number of minutes earlier than T2*GRE images after blood extravasation, as SWI is more sensitive to deoxyhemoglobin than CT scans [3]. The potential of DWI for monitoring disease in the head and neck has shown promising results in patients with head and neck lesions, as shown in recent studies. [4]

Microbleeds have been found to have an association with hypertension, cognitive decline, coronary microvascular disease, stroke, and mortality in different populations. However, somewhat due to differences in scanning techniques, these associations have not been decisively found across all the studies [5-6]. So, sensitive imaging techniques are required to visualize microbleeds, as they predict cerebrovascular events in patients with ischemic stroke, and microbleeds are up to three times more likely to have an ensuing intracerebral hemorrhage (ICH), including hemorrhagic transformation or recurrent ischemic stroke [1].

According to a meta-analysis, microbleeds were presents in 44% of patients with recurrent ischemic stroke and 83% with recurrent ICH [6]. Another recent meta-analysis reported individual patient data pre-thrombolysis, indicating that cerebral microbleeds are independently associated with an increased risk of ICH and poor functional outcome after acute ischemic stroke [7].

So, the most common and alarming complication in an acute ischemic stroke is hemorrhagic transformation, which can significantly worsen prognosis and is usually seen within the first few days following infarction, causing a major decrease in the patient’s quality of life and clinical outcome. The risk of transformation exacerbates exceptionally when considerable cerebral infarctions are present [8-9].

The purpose of this study was to evaluate cerebral microbleeds as a predictor of hemorrhagic transformations, determine the number and location of cerebral microbleeds in an ischemic stroke, and identify if it can be used as a screening tool for a more in-depth assessment and thus intervention.

## Materials and methods

This was a retrospective cohort study of stroke patients. After approval from the institutional review board (IRB) of Shifa International Hospital, Islamabad, data were collected from January 1, 2014, to December 31, 2015.

Microbleeds were visually rated on the SWI scans of the patients by two human raters. Both raters were sufficiently trained prior to the visual analysis. In case of any discordance between the raters, a consultant radiologist was consulted. Follow-up CT/MRI scans were reviewed for hemorrhagic transformations. The number and location of the microbleeds were recorded on the proforma. Data were analyzed with SPSS 21 (IBM Corp., Armonk, NY, US) and the Chi-squared test was applied. Age, sex, pertinent comorbidities, and location were recorded on a standard proforma. The microbleeds were graded as absent (total count, 0), mild (1 to 2), moderate (3 to 10), and severe (>10) [10].

Inclusion criteria

Patients who presented at Shifa International Hospital from January 1, 2014, to December 31, 2015, were reviewed for this study. Among them, patients were included if they presented with acute ischemic stroke and had an SWI/MRI scan within 24 hours of onset and a follow-up CT or MRI scan during present admissions.

Exclusion criteria

Patients were excluded if they had a hemorrhagic stroke or presented after 24 hours of onset of symptoms. Patients were also excluded if they had any previous history of intracerebral hemorrhage.

## Results

**Figure 1:**
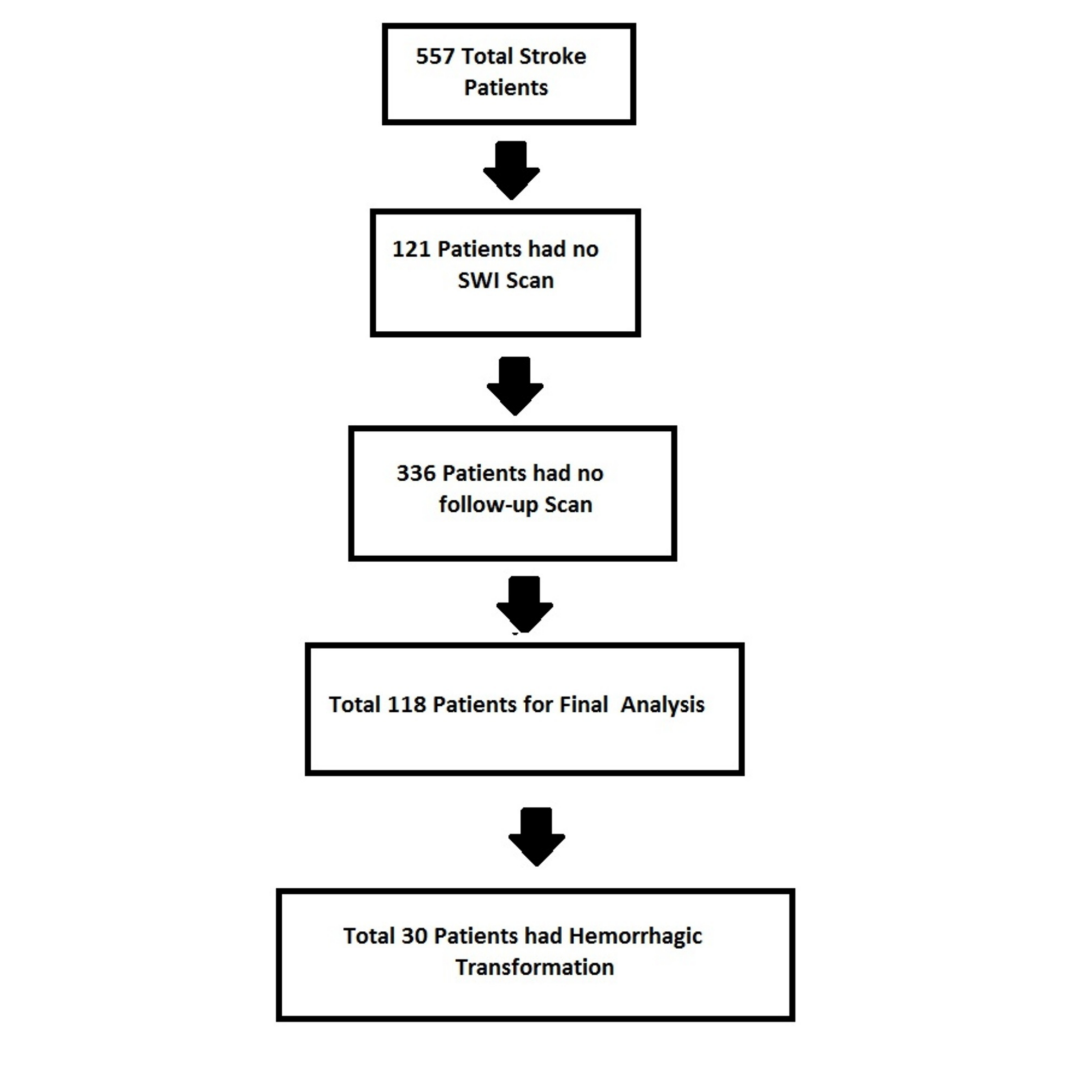
Selection of Patients

During the study period, 575 patients were recorded to have an ischemic stroke, 121 did not have an SWI scan on presentation, and 336 stroke patients had no follow-up scan. A total of 118 patients were included for final analyses (Figure 1). Total 25.4% ischemic stroke patients with microbleeds had a hemorrhagic transformation.

Of these, 63.6% (n=75) were male ischemic stroke patients with microbleeds, out of which 25.3% (n=19) patients had a hemorrhagic transformation, and 36.4% (n=43) were female, out of which 25.6 % (n=11) had a hemorrhagic transformation. On analyzing the age of patients, we found that most hemorrhagic transformations were seen between the age group of 55-64 (n=15, 41.7%), 21% in more than 65, 18.2% in 45-54, and 7.1% in the less than 45 age group. We did not find any statistical co-relation of co-morbidities with hemorrhagic transformations but most of our patients with microbleeds were diabetic and hypertensive with transformation as follows, diabetes (25.4%), hypertension (27.8), high body mass index (BMI, 19.1%), heart diseases (24%), smoking (19%), hyperlipidemia (15%) but no significant values were found. The use of anticoagulants had no statistical significant effect on hemorrhagic transformation, 31.8% had transformations with anticoagulant use, while 23.9 % had transformations without anticoagulant use (Table 1).

**Table 1:** Transformation of Microbleeds with Patient Characteristics BMI: Body Mass Index

Characteristics		Yes	No
Age group	Less than 45	1	13
	45-54	2	9
	55-64	15	21
	More than 65	12	45
Gender	Male	19	56
	Female	11	32
Comorbidities	Diabetes	16	47
	Hypertension	25	65
	High BMI	9	38
	Heart disease	12	38
	Smoking	4	17
	Hyperlipidemia	5	28
Anticoagulant use	Yes	7	15
	No	23	73

While comparing the locations of microbleeds, we found a statistically significant association of the conversion of ischemic stroke to hemorrhage in 45.6% of patients with microbleeds in the parietal region (n=21) with a p-value of <0.001), with transformations in the basal ganglia (35.39%), temporal (33.3%), infratentorial (25%), and frontal (20%). On analyzing different grades of microbleeds, we found a significant statistical association of microbleeds with hemorrhagic transformation. We found that as the grade of microbleeds increases, the chances of conversion of ischemic strokes to hemorrhages increase with a significant p-value of <0.001. In grade four (>10 MB), there was a 73.3% transformation of ischemic stroke to hemorrhage, in grade three 32.6%, in grade two 7.8%. On comparing different size of microbleeds, we found that as the size of microbleeds increases, there were more chances of the conversion of ischemic stroke to hemorrhage, with a significant p-value of 0.001. For 0-1.9 mm, there were 1.5% hemorrhagic transformations; for 2-2.9 mm, 14.6% transformation; for 3-3.9 mm, the transformations were 40.6%; and with 4-5 mm, the transformation was 53.3% (Table 2).

**Table 2:** Transformation of Microbleeds with Different Microbleed Characteristics

Characteristics		Yes	No
Locations	Parietal	21	25
	Frontal	5	21
	Basal Ganglia	14	25
	Occipital	5	6
	Frontal	15	30
	Infratentorial	16	18
Grades	Grade 1	0	6
	Grade 2	4	47
	Grade 3	15	31
	Grade 4	11	4
Size	None	0	5
	0-1.9 mm	3	21
	2-2.9 mm	6	35
	3-3.9 mm	13	19
	4-4.9 mm	8	5
	5-6 mm	0	3

**Figure 2:**
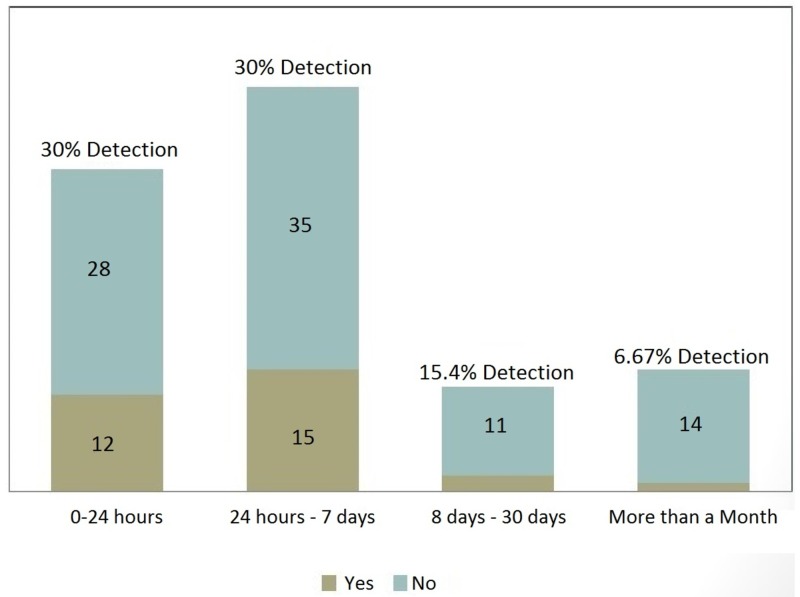
Detection of Microbleeds

The detection of a hemorrhagic transformation was possible in 30% within the first 24 hours, 30% within 24 hours to seven days, 15.4% within eight to 30 days, and 6.67% in more than 30 days (Figure 2).

## Discussion

Numerous studies have shown the association of the presence of microbleeds with cognitive decline along with it being a reputed marker of future stroke risk [1]. Developments in neuroimaging have led to a greater understanding of pathophysiology and the risk of intracerebral hemorrhage (ICH) [11]. It was found that despite SWI allowing the identification of approximately twice as many lesions as T2* GRE, this did not change the clinical associations in multivariate analyses [12].

So, we focused our study on SWI imaging and the identification of microbleeds in ischemic stroke patients and on hemorrhagic transformations. We included patients who had an SWI scan done within 24 hours of an ischemic stroke and had a follow-up CT or MRI scan during the stay. Two team members recorded the data on a self-made proforma, keeping the criteria in mind. They recorded the patient’s information, keeping the patients' name confidential, comparing both the scans, and analyzing the data on SPSS.

In our study, 63.6% were male and 33.4% were female ischemic stroke patients with microbleeds but the hemorrhagic transformations were 25.3% and 25.6% in both genders.

Age has been associated with the presence of microbleeds (P<0.001) on an SWI scan according to studies [12], with microbleeds being more frequent with increasing age, from 6% in those aged 45–50 years to 36% in those aged 80 or more [13]. In our study, we found that hemorrhagic transformations were seen most between the age groups of 55-64 (41.7%) and 21% in more than 65 while less transformation was seen in the age group of less than 45-55 (18.2%) and even less in the less than 45 age group (7.1%). Thus, patients with increasing age and microbleeds on an SWI scan should be taken as a risk for hemorrhagic transformation in case of an ischemic stroke.

Increase in the number of microbleeds (grade) showed a statistical association with hemorrhagic transformation with a p-value <0.001. Grade three showed a 32.6% transformation and grade four showed a 73.3% transformation. Similar results in a study done in 2010 showed an association with a greater baseline number of microbleeds and ICH-related mortality. It also concluded that the risk of ICH-related mortality may outweigh the potential benefit of antithrombotic therapy in individuals with multiple (≥five) microbleeds (adjusted RR 2.5–6%) [14].

The location, grade, and size of the microbleed showed statistical significance in patients for hemorrhagic transformation with a p-value of <0.001 and 0.001 while co-morbidities and the use of anticoagulants showed no statistical association for hemorrhagic transformation.

The detection of hemorrhagic transformation in ischemic stroke patients with microbleeds in the first 24 hours or 24 hours to seven days is a very important clinical factor and a determinant for hemorrhagic transformation that can help a physician to treat patients on time for a better clinical outcome that will improve his quality of life. Another study described the association between hemorrhage burden and a composite endpoint, including death, any loss of function, or cognitive impairment patients with ICH [15].

Our study has some limitations. As we only used SWI scans for the detection of microbleeds, no comparison was made with other scans for detection. Secondly, as the patients' data used consists of patients presenting to our facility only, so a much broader study is needed.

## Conclusions

Only identified relatively recently, microbleeds are increasingly appreciated as a marker of underlying disease states and a risk for ischemic and hemorrhagic sequelae and cognitive decline. Microbleeds detected on an SWI scan are a relevant and accurate predictor of hemorrhagic transformations in acute ischemic infarcts and should be added to MRI stroke protocols for the better management and prediction of outcomes.
